# Patterns in Nonopioid Pain Medication Prescribing After the Release of the 2016 Guideline for Prescribing Opioids for Chronic Pain

**DOI:** 10.1001/jamanetworkopen.2022.16475

**Published:** 2022-06-10

**Authors:** Jason E. Goldstick, Gery P. Guy, Jan L. Losby, Grant T. Baldwin, Matthew G. Myers, Amy S. B. Bohnert

**Affiliations:** 1 Injury Prevention Center, University of Michigan, Ann Arbor; 2 Department of Emergency Medicine, University of Michigan, Ann Arbor; 3 Division of Overdose Prevention, Centers for Disease Control and Prevention, Atlanta, Georgia; 4 Department of Anesthesiology, University of Michigan, Ann Arbor; 5 Veterans Affairs Center for Clinical Management Research, Ann Arbor, Michigan

## Abstract

**Importance:**

In 2016, the Centers for Disease Control and Prevention (CDC) released the evidence-based Guideline for Prescribing Opioids for Chronic Pain. How the release of this guideline coincided with changes in nonopioid pain medication prescribing rates remains unknown.

**Objective:**

To evaluate changes in nonopioid pain medication prescribing after the 2016 CDC guideline release and to assess the heterogeneity in these changes as a function of patient demographic and clinical characteristics.

**Design, Setting, and Participants:**

This cohort study constructed 7 (4 preguideline and 3 postguideline) annual cohorts using claims data from the national Optum Clinformatics Data Mart Database for the period January 1, 2011, through December 31, 2018. The cohorts included adults with commercial insurance, no cancer or palliative care claims, and 2 years of continuous insurance enrollment. Individuals could qualify for inclusion in multiple cohorts. Each cohort covered a 2-year period, with year 1 as the baseline period used to calculate opioid exposure and other clinical characteristics and year 2 as the follow-up period used to calculate prescribing outcomes. Data were analyzed in March 2022.

**Exposures:**

The CDC guideline, which was released in March 2016.

**Main Outcomes and Measures:**

The primary outcome was receipt of any nonopioid pain medication prescriptions (analgesics or antipyretics, anticonvulsants, antidepressants, and nonsteroidal anti-inflammatory drugs) during the follow-up period. This postguideline prescribing pattern was compared with estimates based on the preguideline prescribing pattern, and then the differences were stratified by patient clinical characteristics (chronic pain, recent opioid exposure, substance use disorder, anxiety disorder, and mood disorder).

**Results:**

A total of 15 879 241 individuals (2015 mean [SD] age, 50.2 [18.6] years; 8 298 271 female patients [52.3%]) qualified for inclusion in 1 or more cohorts. Logistic regression models showed that nonopioid pain medication prescribing odds were higher by 3.0% (95% CI, 2.6%-3.3%) in postguideline year 1, by 8.7% (95% CI, 8.3%-9.2%) in postguideline year 2, and by 9.7% (95% CI, 9.2%-10.3%) in postguideline year 3 than the preguideline pattern–based estimates. The magnitude of the postguideline departures from the preguideline pattern varied by several clinical characteristics (chronic pain, recent opioid exposure, anxiety disorder, and mood disorder). The largest departure was found among those with chronic pain, with postguideline prescribing being higher than estimated in postguideline year 2 (13.6%; 95% CI, 12.7%-14.6%) and postguideline year 3 (14.9%; 95% CI, 13.8%-16.0%).

**Conclusions and Relevance:**

Results of this study showed increases in nonopioid pain medication prescribing after the release of the 2016 CDC guideline, suggesting that the guideline may be associated with an increase in guideline-concordant care, but additional studies are needed to understand the role of other secular changes in the opioid policy landscape and other sources of nonopioid medication use.

## Key Points

**Question:**

Did nonopioid pain medication prescribing change after the release of the Centers for Disease Control and Prevention Guideline for Prescribing Opioids for Chronic Pain?

**Findings:**

In this cohort study of the insurance claims data of more than 15 million patients, nonopioid pain medication prescribing rates were higher by 3.0% in postguideline year 1, by 8.7% in postguideline year 2, and by 9.7% in postguideline year 3 than the preguideline pattern–based estimates. All patient subpopulations analyzed showed larger-than-expected prescribing rates, but the relative magnitude of the difference varied.

**Meaning:**

Findings of this study suggest that the 2016 guideline may have been a factor associated with increased rates of nonopioid pain medication prescribing.

## Teaser

This cohort study examines the preguideline and postguideline prescribing rates of nonopioid vs opioid pain medication among individuals with commercial insurance.

## Introduction

The drug overdose epidemic has greatly affected the US in the past 2 decades both in terms of mortality (with 92 000 deaths in 2020 and high provisional estimates in 2021)^1,2^ and associated harms, such as opioid use disorder.^3^ Although opioid overdose mortality dynamics have shifted during this period,^4,5,6^ prescription opioids have remained a critical factor in opioid-related morbidity and mortality.^7,8,9^ Recognizing the need for evidence-based guidelines for safe opioid prescribing, the Centers for Disease Control and Prevention (CDC) released the Guideline for Prescribing Opioids for Chronic Pain^10^ in March 2016.

The 2016 CDC guideline provides evidence-based recommendations for opioid prescribing and use in the treatment of chronic pain among adult patients (18 years or older) in outpatient primary care settings, excluding those receiving care for cancer and palliative and end-of-life care. The guideline emphasizes that patients with chronic pain should receive care that provides the greatest benefit; for example, initiating opioids only when the expected benefits outweigh the risks, and providing nonopioid pain treatments instead of, or in addition to, opioids. Thus, guideline-concordant care may be reflected, in part, by increases in nonopioid pain medication prescribing after the guideline release.

Evidence suggests that specific elements of the 2016 CDC guideline are associated with changes in opioid prescribing,^11,12,13,14^ but no study has focused on corresponding changes in nonopioid prescribing. Specifically, evidence suggests that opioid prescribing decreased after the guideline was released,^11^ and 1 analysis showed that this decline primarily manifested through an acceleration of the preexisting decreasing pattern.^12^ Acceleration of previous decreases in opioid prescribing was corroborated in another study that showed lower dose and duration of initial opioid prescriptions among patients who were opioid naive.^13^ Focusing on unintended consequences of the guideline, such as rapid tapering,^15^ other analyses found that, among patients receiving long-term opioid therapy, increased incidence of rapid tapering coincided with the guideline release.^14^ One purpose of the guideline is to improve the safety and effectiveness of pain treatment; thus, given the evidence that opioid prescribing decreased, there may be a corresponding increase in uptake of nonopioid pharmacologic and nonpharmacologic pain relief.^16,17^ Previous research has reported higher rates in both opioid and nonopioid prescribing to patients with HIV,^18^ relatively static rates of nonopioid pain medication prescribing,^19^ and variability across clinician specialty in the tendency to prescribe opioids.^20,21^ None of those studies, however, featured time frames both before and after the guideline release.

In this cohort study, we evaluated changes in nonopioid pain medication prescribing, including acetaminophen, nonsteroidal anti-inflammatory drugs (NSAIDs), antidepressants, and antiseizure medications (all of which are evidence-based pain therapies^22,23^) after the 2016 CDC guideline release and how those changes compared with the changes expected from the preexisting secular pattern. We also assessed the heterogeneity in prescribing changes as a function of patient demographic and clinical characteristics. We hypothesized that nonopioid pain medication prescribing would increase after the guideline release and would be above and beyond the preexisting pattern–based expectations.

## Methods

The University of Michigan Institutional Review Board deemed this cohort study nonregulated; thus, it was exempt from review, and the informed consent requirement was waived because we did not have access to patient-identifying information. We followed the Strengthening the Reporting of Observational Studies in Epidemiology (STROBE) reporting guideline.

### Data Source

We used the Optum Clinformatics Data Mart Database as the primary data source. The database contains records of all medical services billed to a large, national insurer, including employer-sponsored insurance and Medicare Advantage, for both medical procedures and pharmacy costs. Those medical records include claims made in all 50 US states, the District of Columbia, and Puerto Rico. In addition to state or territory, limited patient-level demographic data (age, sex, and race and ethnicity) were available. Race and ethnicity data were reported in the database. The following categories were identified: Asian, Black, Hispanic, White, and missing or unknown. For either cohort construction or outcome measurements, we used data from January 1, 2011, through December 31, 2018.

### Cohort Definitions

To evaluate year-to-year changes in prescribing, we constructed sequential cohorts. Each cohort covered a 2-year period: year 1 consisted of a baseline period used to identify opioid exposure and other clinical characteristics, and year 2 consisted of a follow-up period used to measure the prescribing outcome data (eFigure 1 in the Supplement). The inclusion requirement for each cohort was continuous insurance enrollment in that 2-year period, and exclusion requirements were age younger than 18 years and cancer diagnosis or palliative care during the 2-year period; the number of patients excluded for each reason is shown in eFigure 2 in the Supplement.

In total, there were 7 cohorts. The follow-up period was before the 2016 CDC guideline release for 4 cohorts (cohorts 1-4) and after the release for 3 cohorts (cohorts 5-7). Individuals could qualify for inclusion in multiple cohorts.

### Clinical Measurements

The primary outcome was receipt of any nonopioid pain medication prescriptions during follow-up. The 4 primary categories of nonopioid pain medications were NSAIDs, analgesics or antipyretics, select anticonvulsants used to treat pain, and select antidepressants used to treat pain. We used a list of drugs from a previous study^24^ (eTable 1 in the Supplement). Because of the difficulties of quantifying dose, particularly across categories, we analyzed a binary indicator of receipt of any nonopioid pain medication prescription during follow-up.

To stratify the patient population into those with vs those without recent opioid exposure, we calculated an indicator of receipt of any opioid prescription during the 12-month baseline period. Opioid pain medications were ascertained using the CDC morphine milligram equivalent conversion table (drugs are listed in eTable 2 in the Supplement).^25^ For descriptive comparisons, we also calculated a binary indicator of receipt of any opioid prescription during follow-up.

To calculate an indicator of chronic pain during baseline, we indexed inpatient and outpatient records using *International Classification of Diseases, Ninth Revision* and *International Statistical Classification of Diseases and Related Health Problems, Tenth Revision* diagnosis codes for back or neck pain, nonmigraine headaches, osteoarthritis, and fibromyalgia (eTable 3 in the Supplement), as was performed in previous work.^26,27^ Patients with back or neck pain and/or headache pain records had to have received 2 related diagnoses at least 90 days apart from each other within the baseline year to be considered as patients with chronic pain.

Given their potential importance to pain medication prescribing decisions, we included indicators of any substance use disorder (SUD) diagnosis, anxiety disorder diagnosis, and mood disorder (eg, depression) diagnosis. We calculated those indicators from the presence or absence of relevant *International Classification of Diseases, Ninth Revision* and *International Statistical Classification of Diseases and Related Health Problems, Tenth Revision* diagnosis codes in the baseline period (eTable 3 in the Supplement), as were used in previous studies.^28^

### Statistical Analysis

We began the analysis by describing the basic demographic characteristics of each cohort. Next, we descriptively compared the percentage of patients who received opioid vs nonopioid pain medication prescriptions during follow-up, stratified by recent opioid exposure status. We then compared the observed rates of nonopioid pain medication prescribing after the 2016 CDC guideline release with the expected rates of prescribing had the preguideline pattern continued. We performed this comparison by fitting an unadjusted linear probability model to the preguideline prescribing pattern and projecting the model forward to the postguideline period. We presented those differences visually overall and stratified by clinical conditions (opioid exposure, chronic pain, SUD, anxiety disorder, and mood disorder). In tables, we displayed the absolute difference in the percentage of patients who received nonopioid pain medication prescriptions vs the estimated percentage using the linear probability model across demographic and clinical subgroups.

We used logistic regression models to derive the adjusted associations of the 2016 CDC guideline release with the probability of receiving nonopioid pain medication prescriptions during follow-up. These models included fixed-effect variables for state or territory, and thus the adjusted associations could not be confounded by between-state differences. Other control variables included age, sex, race and ethnicity (obtained from the Optum Clinformatics Data Mart database, which used the following categories: Asian, Black, Hispanic, White, and missing or unknown), insurance type, SUD and mental health diagnosis indicators, chronic pain status, and recent opioid exposure. Race and ethnicity were used only as a control variable, not as a basis for stratification.

We included a linear slope for time and used categorical indicators for each postguideline time point (cohorts 5-7) to quantify the departure from that (logistic) linear pattern; significant differences represented the adjusted estimates of the departure from the preguideline pattern, which was the key inferential target of this analysis. We conducted these adjusted analyses overall and stratified them by chronic pain status, recent opioid exposure, and each mental health and SUD diagnosis indicator.

Stastistical tests were 2-sided, and α = .05 indicated statistical significance. Data were analyzed in March 2022 using R, version 3.6.0 (R Foundation for Statistical Computing).

## Results

A total of 15 879 241 patients (2015 mean [SD] age, 50.2 [18.6] years; 8 298 271 female patients [52.3%] and 7 579 557 male patients [47.7%]; missing data for 1413 patients) qualified for inclusion in 1 or more cohorts. A cohort ranged in size from 5 408 548 (cohort 3, which covered January 1, 2013, to December 31, 2014) to 7 610 075 (cohort 7, which covered January 1, 2017, to December 31, 2018). The demographic characteristics of the 7 cohorts are shown in Table 1, and their clinical characteristics are shown in Table 2. The percentage of White patients and the percentage of patients with commercial insurance decreased each year, whereas the mean age increased. The proportion of patients with chronic pain, SUD, anxiety disorder, and mood disorder increased each year, whereas the proportion with past-year opioid exposure decreased.

**Table 1.** Demographic Characteristics of the Cohorts, From 2011 to 2018

Cohort	No. of patients	Female sex, No. (%)^a^	Male sex, No. (%)^a^	White race and ethnicity, No. (%)^b^	Age, mean (SD), y	Commercial insurance coverage, No. (%)
1	5 755 311	3 038 883 (52.8)	2 715 505 (47.2)	3 867 789 (67.2)	49.1 (17.5)	4 352 226 (75.6)
2	5 941 176	3 127 795 (52.6)	2 812 503 (47.3)	3 980 412 (67.0)	49.7 (17.8)	4 333 082 (72.9)
3	5 408 548	2 810 919 (52.0)	2 596 891 (48.0)	3 604 325 (66.6)	50.2 (18.1)	3 833 344 (70.9)
4	5 648 410	2 943 617 (52.1)	2 704 213 (47.9)	3 702 536 (65.6)	50.4 (18.2)	3 953 624 (70.0)
5	6 150 982	3 238 238 (52.7)	2 912 304 (47.3)	4 023 768 (65.4)	51.6 (18.5)	4 118 237 (67.0)
6	6 893 796	3 641 623 (52.8)	3 251 852 (47.2)	4 380 502 (63.5)	52.6 (18.7)	4 353 309 (63.1)
7	7 610 075	4 035 949 (53.0)	3 573 791 (47.0)	4 646 201 (61.1)	53.6 (18.8)	4 609 204 (60.6)

^a^ There were 4215 missing values across years in the sex variable and were included in the denominator.

^b^ Unknown values (approximately 10% across years) in the race and ethnicity variable were included in the denominator when calculating the percentage of White patients. Race and ethnicity data were reported in the Optum Clinformatics Data Mart Database and included the following categories: Asian, Black, Hispanic, White, and missing or unknown. Only the White group is included in this table because it was the largest; the other groups were not included because of space constraints.

**Table 2.** Clinical Characteristics of the Cohorts at Baseline, From 2011 to 2018

Cohort	No. of patients	Opioid exposure, No. (%)	Chronic pain, No. (%)	Substance use disorder, No. (%)	Anxiety disorder, No. (%)	Mood disorder, No. (%)
1	5 755 311	1 325 452 (23.0)	952 680 (16.6)	77 817 (1.4)	362 488 (6.3)	477 471 (8.3)
2	5 941 176	1 354 674 (22.8)	1 026 348 (17.3)	86 647 (1.5)	410 550 (6.9)	512 039 (8.6)
3	5 408 548	1 201 900 (22.2)	939 428 (17.4)	85 993 (1.6)	394 035 (7.3)	480 302 (8.9)
4	5 648 410	1 196 785 (21.2)	988 546 (17.5)	97 795 (1.7)	431 554 (7.6)	513 355 (9.1)
5	6 150 982	1 257 374 (20.4)	1 134 682 (18.4)	118 160 (1.9)	528 752 (8.6)	588 679 (9.6)
6	6 893 796	1 416 025 (20.5)	1 345 654 (19.5)	145 568 (2.1)	690 767 (10.0)	696 058 (10.1)
7	7 610 075	1 470 354 (19.3)	1 564 972 (20.6)	170 512 (2.2)	818 596 (10.8)	813 041 (10.7)

### Opioid and Nonopioid Prescribing Patterns

**Figure 1.**
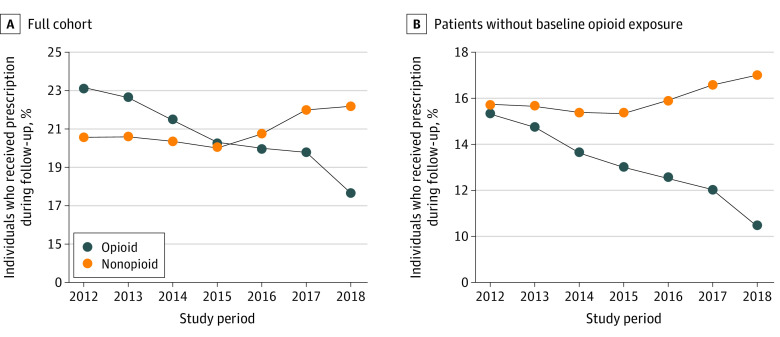
Percentage of Patients Who Received Opioid vs Nonopioid Pain Prescriptions During Follow-up

Figure 1 shows the percentage of patients who received opioid vs nonopioid pain medication prescriptions during follow-up, both among the full cohort and only patients with recent opioid exposure. Among the full cohort, the percentage of patients who received opioid prescriptions decreased every year by nearly one-third across the study period (23.1% in 2012 and 17.6% in 2018), whereas nonopioid pain medication prescriptions remained steady through 2015 but increased each year thereafter (20.1% in 2015 and 22.2% in 2018). Among only patients without recent opioid exposure, the percentage who received nonopioid vs opioid pain medication prescriptions during follow-up was nearly the same in 2012 (15.7% vs 15.3%) but diverged further each year thereafter until the percentage who received nonopioid pain medication prescriptions was more than 50% greater than that of patients who received opioid prescriptions in 2018 (17.0% vs 10.5%).

**Figure 2.**
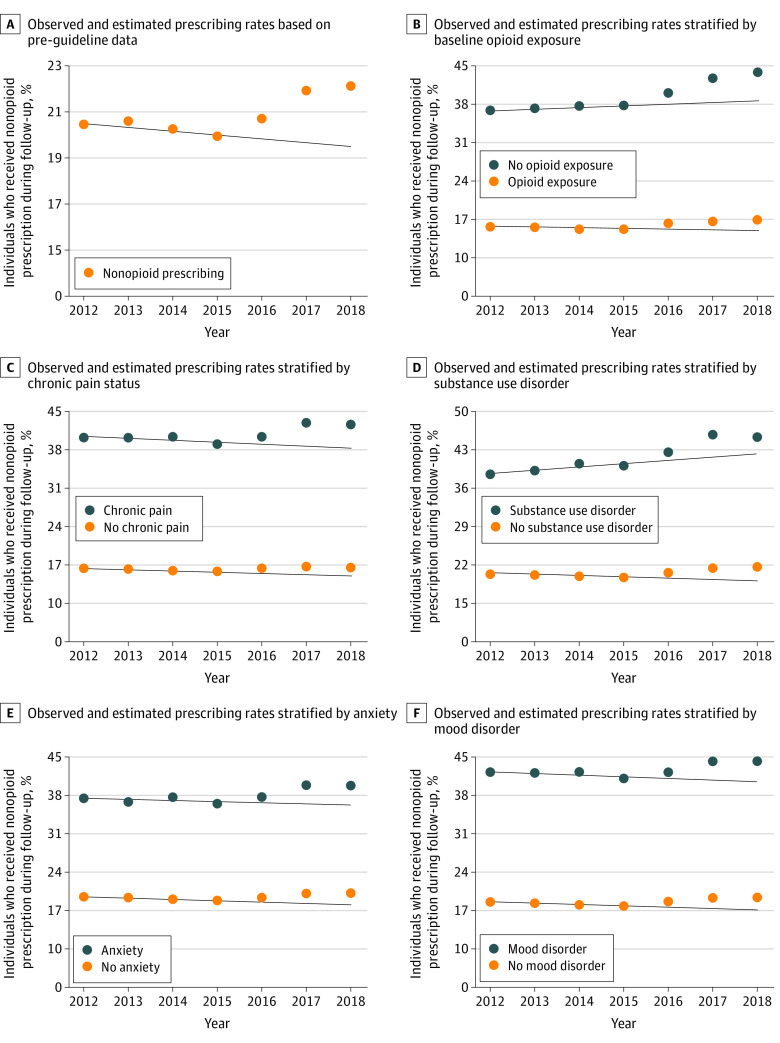
Observed vs Estimated Nonopioid Medication Prescribing Rates The solid lines are linear estimates based on the preguideline prescribing patterns in the given subpopulation.

The percentage of patients who received nonopioid pain medication prescriptions in each cohort and were stratified by demographic and clinical characteristics are shown in eTables 4 and 5 in the Supplement, respectively. Nonopioids were more likely to be prescribed to women than men, those with Medicare Advantage vs commercial insurance, and those with (vs without) all 5 clinical characteristics (recent opioid exposure, chronic pain, SUD, anxiety disorder, and mood disorder). For example, those with chronic pain consistently had more than double the rates of nonopioid pain medication prescribing than those without chronic pain (cohort 1: 40.4% vs 16.7%; cohort 7: 42.7% vs 16.9%). The percentage of patients who received nonopioid pain medication prescriptions during follow-up was greater after the 2016 CDC guideline release than the preguideline pattern–based estimates in every patient subpopulation analyzed, although the magnitude of the difference varied. In addition, the departure between the observed and estimated prescribing rates was statistically significantly greater in cohort 6 (2.1% higher) and cohort 7 (2.5% higher) than in cohort 5 (0.8% higher), which was the first postguideline cohort, except among patients with commercial insurance, for whom the departures were more similar in cohort 5 (1.0% higher), cohort 6 (1.3% higher), and cohort 7 (1.8% higher). The patterns in the observed vs estimated prescribing rates stratified by the 5 clinical characteristics are shown in Figure 2.

### Adjusted Analysis of Nonopioid Prescribing Patterns

Table 3 shows the adjusted logistic regression of nonopioid pain medication prescribing probability over time in the overall population and stratified by the 5 clinical characteristics. In the overall population, the odds of receiving nonopioid pain medications were higher by 3.0% (95% CI, 2.6%-3.3%) in postguideline year 1, by 8.7% (95% CI, 8.3%-9.2%) in postguideline year 2, and by 9.7% (95% CI, 9.2%-10.3%) in postguideline year 3 than the expected odds based on the preguideline pattern. There were greater increases in prescribing odds in every postguideline year than the preguideline pattern–based estimates among those with (vs without) opioid exposure, and none of the 95% CIs overlapped (eg, postguideline year 3: 12.0% [95% CI, 11.0%-13.0%] vs 9.2% [95% CI, 8.6%-9.9%]) (Table 3). Similarly, those with chronic pain, anxiety disorder, and mood disorder compared with those without these conditions showed larger relative increases in prescribing odds, and none of the 95% CIs in postguideline years 2 and 3 overlapped. For example, in postguideline year 3, those with chronic pain had a prescribing rate that was 14.9% (95% CI, 13.8%-16.0%) higher than estimated from the preguideline pattern, whereas those without chronic pain had only an 8.7% (95% CI, 8.0%-9.3%) higher-than-estimated rate based on the preguideline pattern. There was a smaller difference between those with and those without SUD, and the 95% CIs for the relative increase overlapped in every postguideline year (eg, postguideline year 2: 10.8% (95% CI, 7.8%-13.8%) vs 8.7% (95% CI, 8.2%-9.1%) (Table 3).The largest departure was found among those with chronic pain, with postguideline prescribing being higher than estimated in postguideline year 2 (13.6%; 95% CI, 12.7%-14.6%).

**Table 3.** Logistic Regression Model for the Receipt of Nonopioid Pain Prescriptions During Follow-up^a^

Characteristic	Odds ratio (95% CI)		
	Postguideline year 1	Postguideline year 2	Postguideline year 3
Overall	1.03 (1.03-1.03)	1.09 (1.08-1.09)	1.10 (1.09-1.10)
Opioid exposure			
With	1.06 (1.05-1.06)	1.11 (1.10-1.12)	1.12 (1.11-1.13)
Without	1.02 (1.02-1.02)	1.08 (1.08-1.09)	1.09 (1.09-1.10)
Chronic pain			
With	1.04 (1.03-1.05)	1.14 (1.13-1.15)	1.15 (1.14-1.16)
Without	1.03 (1.03-1.03)	1.08 (1.07-1.08)	1.09 (1.08-1.09)
Substance use disorder			
With	1.05 (1.02-1.07)	1.11 (1.08-1.14)	1.10 (1.07-1.14)
Without	1.03 (1.03-1.03)	1.09 (1.08-1.09)	1.10 (1.09-1.10)
Anxiety disorder			
With	1.04 (1.03-1.05)	1.13 (1.11-1.14)	1.13 (1.12-1.15)
Without	1.03 (1.03-1.03)	1.08 (1.08-1.09)	1.09 (1.09-1.10)
Mood disorder			
With	1.04 (1.03-1.05)	1.12 (1.10-1.13)	1.13 (1.11-1.14)
Without	1.03 (1.03-1.03)	1.08 (1.08-1.09)	1.09 (1.09-1.10)

^a^ The entries quantify the relative difference in the odds of receiving nonopioid pain prescriptions after the release of the Centers for Disease Control and Prevention Guideline for Prescribing Opioids for Chronic Pain compared with the logistic-linear time pattern before the guideline was released. All clinical characteristics were ascertained during the 12-month baseline period. All models controlled for state, age, sex, race and ethnicity, insurance type, chronic pain, baseline opioid exposure, substance use disorder, anxiety disorder, and mood disorder. In the stratified models, the stratifying variable was excluded from the list of control variables because of redundancy.

## Discussion

We found evidence of increased nonopioid pain medication prescribing after the 2016 CDC guideline release, rates that were above and beyond the expectation based on the preexisting pattern. To our knowledge, this study was the first to analyze patterns in nonopioid pain medication prescribing, providing important context to other analyses that reported changes in opioid prescribing after the release of the guideline.^11,12,13^ Increases in nonopioid pain medication prescribing were robust across all patient subpopulations analyzed and coincided with significant decreases in opioid prescribing presented descriptively in the present study and documented in previous research.^11,12^ In addition, there was evidence that the postguideline trajectory departed more from the preguideline pattern–based estimates among patients with recent opioid exposure, chronic pain, and anxiety disorder and/or mood disorder. These findings are consistent with clinicians prescribing nonopioid medications for pain to a greater extent currently than before the release of the guideline. Moreover, this change is consistent with the first recommendation of the guideline to prioritize nonopioid pain therapies when it is not clear whether the risks of opioids outweigh their benefits.

This cohort study found that pain medication prescribing practices for those with recent opioid exposure changed substantially from 2012 to 2018. Specifically, in 2012, the prevalence of opioid prescribing among those with recent opioid exposure was approximately equal to the prevalence of nonopioid pain medication prescribing; by 2018, the prevalence of nonopioid prescribing was more than 50% higher in the same group. This finding aligns with language in the 2016 CDC guideline advising caution when initiating opioids and avoiding their use as a default first-line therapy. Yet, given that the decreases in opioid prescribing were not completely offset by the increases in nonopioid prescribing, further research is needed to ascertain whether there were commensurate increases in nonpharmacologic pain treatment. Future studies are also needed to identify potential undertreatment of pain across all forms of treatment.

Increased postguideline nonopioid prescribing beyond that expected from the preguideline pattern was found across all clinical subsets analyzed, although the increases were larger in some patient subpopulations. These increases among those with chronic pain and recent opioid exposure are consistent with previous reports of changes to prescriptions among patients on long-term opioid therapy, many of whom had chronic pain, and suggest possible substitution of opioid with nonopioid medications.^14^ Larger increases among those with anxiety and mood disorders may be associated with these patients being generally more likely to be prescribed opioids,^29,30^ and the postguideline nonopioid prescribing changes may signal a greater awareness of the risks of opioid use disorder.

Across all patient populations, to optimize pain management and ensure fidelity to the purpose of the 2016 CDC guideline, care must be taken to avoid misapplication of the recommendations.^15^ For example, practices such as rapid tapering and switching of medications from a stable, long-term opioid dose in the absence of harms are not consistent with the language of the guideline. Patients may find tapering and medication switching to be challenging and could encounter serious risks associated with withdrawal symptoms, increased pain, or unrecognized opioid use disorder, which must be accounted for in patient-centered clinical decisions.^15^ Given the heterogeneity of the effectiveness of nonopioid pain medications for acute^31^ and chronic^23,32^ pain, the selection of nonopioids for pain treatment should be a collaborative decision between the clinician and patient.

Although there were differences in the baseline rates of nonopioid pain medication prescribing, there was a consistent signal across patient subpopulations of greater departure from the preguideline trajectory as time passed. Overall, women and patients with Medicare Advantage; recent opioid exposure; and chronic pain, SUD, and anxiety or mood disorder were more likely to receive nonopioid pain medication prescriptions across cohorts. There was some heterogeneity in relative magnitude, but almost all patient groups experienced larger reductions in nonopioid pain medication prescribing compared with the preexisting pattern in postguideline year 2 than year 1 and in postguideline year 3 than year 2. That consistent pattern may reflect greater awareness and uptake of guideline recommendations over time, improved implementation of the recommendations in subsequent years, or other secular patterns, such as differing prescribing norms brought about by the guideline. Future research is needed to examine how these patterns progress or level off to ascertain whether guideline modifications, or changes in messaging and implementation, are associated with further beneficial increases in nonopioid pain medication prescribing.

We believe this cohort study provides new insight into changes in pharmacologic nonopioid pain treatment after the 2016 CDC guideline release, but further work is required to clarify this finding and to explore other changes that are associated with guideline uptake, including shift to the use of some nonpharmacologic treatment modalities. Given the documented differences in the propensity for opioid prescribing across medical specialties,^20^ analyses of the prescribing changes by specialty and by geography (eg, regional and urban vs rural contrasts)^33^ could identify additional areas for improvement. This work and previous evaluations of postguideline changes in opioid prescribing^12,13^ may be supplemented by studies of changes in nonpharmacologic pain therapy uptake, undertreatment of pain, and patient pain outcomes, which may present a fuller picture of the evolution of the pain management landscape.

Health system interventions, such as quality improvement efforts and measures, care coordination, electronic health record–based clinical decision supports, and data dashboards, may support and sustain practice improvements. Multimodal and multidisciplinary approaches to pain management that address the biological, psychological, and social characteristics of each person are a critical part of a comprehensive treatment protocol. Further analyses can help identify (1) the fidelity to that goal, (2) the long-term comparative effectiveness of different treatment modalities, and (3) the avenues through which pain relief can be optimized.

### Limitations

This study has several limitations. First, because the analysis used insurance claims data from a single national insurer, it focused on a subset of the US population with relatively high socioeconomic status, particularly given the continuous insurance enrollment requirement for the cohorts. Second, the study could not capture pain management care sought without coverage from insurance or Medicare Advantage, Medicaid, or a secondary insurance plan, or purchased over the counter. Third, being limited to the information in the medical claims, we potentially missed data on comorbidities that predated the beginning of coverage (eg, SUD and pain or injury), pain intensity, and effectiveness of pain management. Fourth, clinician specialty was not incorporated into this analysis, which is known to be a factor in pain medication prescribing practices.^20^ Fifth, we were unable to measure or incorporate the differences in guideline implementation across regions, institutions, and medical specialties. Sixth, some of the nonopioid pain medications (eg, antidepressants) have nonpain-related uses; however, most of the nonopioid pain medications in these data were NSAIDs, minimizing the impact of this limitation. Relatedly, we did not include certain nonopioid medications that may be used for pain, such as lidocaine, benzodiazapines, and skeletal muscle relaxants, because of insufficient evidence of their use for treating chronic pain.^23^ Seventh, secular patterns in the cohorts (eg, aging) were evident, and, although the primary results adjusted for demographic and clinical characteristics, unmeasured confounding underlying those secular shifts was possible.

## Conclusions

This cohort study found higher-than-expected increases in nonopioid pain medication prescribing after the release of the 2016 CDC guideline, coinciding with reductions in opioid prescriptions. This finding suggests that clinicians have prescribed nonopioid medications for pain more frequently over time and that clinicians have tended to consider opioid therapy only if its expected benefits for both pain and function outweighed its risks to the patient. In addition, these findings suggest that, if opioids are prescribed, they might be prescribed in combination with nonopioid pharmacologic therapy more so than in the past. Although the results of this study suggest that the 2016 CDC guideline may be associated with an increase in guideline-concordant care, additional work is necessary to examine the contributions of other secular changes in the opioid policy landscape and other sources of nonopioid medication use, including nonprescription use. It will also be important to assess other shifts in these patterns after the release of the 2022 guideline.

## Notes

Supplement. **eTable 1.** Generic Drug Names Used in Identifying Non-Opioid Prescriptions **eTable 2.** Generic Names of Opioid Prescriptions Identified by NDC Code Within the 2020 CDC Opioid NDC and Oral MME Conversion File **eTable 3.** ICD-9 & ICD-10 Codes Used to Indicate Clinical Characteristics **eTable 4.** Percent Receiving Non-Opioid Pain Medication Prescriptions During Follow-Up in Each Cohort, and Stratified by Demographic Characteristics; in the Post-Guideline Period (2016-2018) and Differences Between the Observed Rate and That Predicted From the Pre-Guideline Trend **eTable 5.** Percent Receiving Non-Opioid Pain Medication Prescriptions During Follow-Up in Each Cohort, and Stratified by Baseline Clinical Characteristics; in the Post-Guideline Period (2016-2018) and Differences Between the Observed Rate and That Predicted From the Pre-Guideline Trend **eFigure 1.** Schematic Diagram of the Sequential Cohorts **eFigure 2.** Total Number Eligible for Each Cohort, and How Many Were Deleted Due to the Continuous Enrollment Requirement, the Age 18+ Requirement, and the Cancer Exclusion
