# Supplementary material for: Patterns in Nonopioid Pain Medication Prescribing After the Release of the 2016 Guideline for Prescribing Opioids for Chronic Pain
Source: JAMA Netw Open. 2022 Jun 10;5(6):e2216475. doi: 10.1001/jamanetworkopen.2022.16475 (PMC9187961; doi:10.1001/jamanetworkopen.2022.16475)
Supplement: Supplement. — eTable 1. Generic Drug Names Used in Identifying Non-Opioid Prescriptions eTable 2. Generic Names of Opioid Prescriptions Identified by NDC Code Within the 2020 CDC Opioid NDC and Oral MME Conversion File eTable 3. ICD-9 & ICD-10 Codes Used to Indicate Clinical Characteristics eTable 4. Percent Receiving Non-Opioid Pain Medication Prescriptions During Follow-Up in Each Cohort, and Stratified by Demographic Characteristics; in the Post-Guideline Period (2016-2018) and Differences Between the Observed Rate and That Predicted From the Pre-Guideline Trend eTable 5. Percent Receiving Non-Opioid Pain Medication Prescriptions During Follow-Up in Each Cohort, and Stratified by Baseline Clinical Characteristics; in the Post-Guideline Period (2016-2018) and Differences Between the Observed Rate and That Predicted From the Pre-Guideline Trend eFigure 1. Schematic Diagram of the Sequential Cohorts eFigure 2. Total Number Eligible for Each Cohort, and How Many Were Deleted Due to the Continuous Enrollment Requirement, the Age 18+ Requirement, and the Cancer Exclusion [file jamanetwopen-e2216475-s001.pdf]

Supplemental Online Content

Goldstick JE, Guy GP, Losby JL, Baldwin GT, Myers MG, Bohnert ASB. Patterns in nonopioid pain medication prescribing after the release of the 2016 Guideline for Prescribing Opioids for Chronic Pain. *JAMA Netw Open*. 2022;5(6):e2216475. doi:10.1001/jamanetworkopen.2022.16475

eTable 1. Generic Drug Names Used in Identifying Non-Opioid Prescriptions

eTable 2. Generic Names of Opioid Prescriptions Identified by NDC Code Within the 2020 CDC Opioid NDC and Oral MME Conversion File

eTable 3. ICD-9 & ICD-10 Codes Used to Indicate Clinical Characteristics

eTable 4. Percent Receiving Non-Opioid Pain Medication Prescriptions During Follow-Up in Each Cohort, and Stratified by Demographic Characteristics; in the Post-Guideline Period (2016-2018) and Differences Between the Observed Rate and That Predicted From the Pre-Guideline Trend

eTable 5. Percent Receiving Non-Opioid Pain Medication Prescriptions During Follow-Up in Each Cohort, and Stratified by Baseline Clinical Characteristics; in the Post-Guideline Period (2016-2018) and Differences Between the Observed Rate and That Predicted From the Pre-Guideline Trend

eFigure 1. Schematic Diagram of the Sequential Cohorts

eFigure 2. Total Number Eligible for Each Cohort, and How Many Were Deleted Due to the Continuous Enrollment Requirement, the Age 18+ Requirement, and the Cancer Exclusion

This supplemental material has been provided by the authors to give readers additional information about their work.

© 2022 Goldstick JE et al. *JAMA Network Open.*

eTable 1. Generic Drug Names Used in Identifying Non-Opioid Prescriptions

**Analgesics/Antipyretics**	**Nonsteroidal Anti-Inflammatory Drugs**
Acetaminophen	Al Hydroxide/Mg Trisilicate/Phenylbutazone
Acetaminophen/Aspirin/Caffeine	Antipyrine/Benzocaine/Glycerin
Acetaminophen/Butalbital	Aspirin
Acetaminophen/Butalbital/Caffeine	Aspirin/Butalbital/Caffeine
Acetaminophen/Caffeine	Bromfenac Sodium
Acetaminophen/Caffeine/Magnesium Salicylate	Celecoxib
Acetaminophen/Calcium Carbonate	Choline Magnesium Trisalicylate
Acetaminophen/Magnesium Salicylate	Diclofenac
Acetaminophen/Phenyltoloxamine Citrate	Diclofenac Potassium
APAP/Ca Carbonate/Mg Carbonate/Mg Oxide	Diclofenac Sodium
APAP/Caff/Magnesium Sal/Phenyltoloxamine Cit	Diclofenac Sodium/Misoprostol
APAP/Caff/Phenyltoloxamine Cit/Salicylamide	Diflunisal
APAP/Phenyltoloxamine Cit/Salicylamide	Esomeprazole Magnesium/Naproxen
**Anticonvulsants**	Etodolac
Carbamazepine	Fenoprofen Calcium
Gabapentin	Flurbiprofen
Gabapentin Enacarbil	Ibuprofen
Oxcarbazepine<br>Pregabalin	Indomethacin
**Antidepressants**	Indomethacin Sodium
Amitriptyline Hydrochloride	Ketoprofen
Amoxapine	Magnesium Salicylate
Clomipramine Hydrochloride	Meclofenamate Sodium
Desipramine Hydrochloride	Mefenamic Acid
Desvenlafaxine	Meloxicam
Desvenlafaxine Succinate	Nabumetone
Doxepin Hydrochloride	Naproxen
Duloxetine Hydrochloride	Naproxen Sodium
Imipramine Hydrochloride	Naproxen Sodium/Sumatriptan Succinate
Imipramine Pamoate	Oxaprozin
Levomilnacipran Hydrochloride	Oxyphenbutazone
Milnacipran Hydrochloride	Phenylbutazone
Milnacipran Hydrochloride;Milnacipran Hydrochl	Piroxicam
Nortriptyline Hydrochloride	Rofecoxib
	Salsalate
Protriptyline Hydrochloride	Sulindac
Trimipramine Maleate	Tolmetin Sodium
Venlafaxine Hydrochloride	

List of 29,202 NDC codes used to index prescription records available upon request.

© 2022 Goldstick JE et al. *JAMA Network Open.*

eTable 2. Generic Names of Opioid Prescriptions Identified by NDC Code Within the 2020 CDC Opioid NDC and Oral MME Conversion File

Generic Name	
A.p.c. W/codeine	Belladonna Extract/Opium
APAP/Butalbital/Caff/Codeine Phos	Buprenorphine
Acetaminophen/Caffeine/Dihydrocodeine Bitartrate	Buprenorphine Hydrochloride
Acetaminophen/Codeine Phosphate	Buprenorphine/Naloxone
Acetaminophen/Hydrocodone Bitartrate	Buprenorphine/naloxone
Acetaminophen/Oxycodone Hydrochloride	Butorphanol Tartrate
Acetaminophen/Tramadol Hydrochloride	Codeine Phosphate
Acetaminophen/butalbital/codeine Phosphate	Codeine Sulfate
Acetaminophen/caffeine/dihydrocodeine Bitartrate	Dihydrocodeine/apap/caffeine
Acetaminophen/codeine Phosphate	Fentanyl
Acetaminophen/hydrocodone Bitartrate	Fentanyl Citrate
Acetaminophen/meperidine Hydrochloride	Hydrocodone Bitartrate
Acetaminophen/oxycodone Hydrochloride	Hydrocodone Bitartrate/Ibuprofen
Acetaminophen/pentazocine Hydrochloride	Hydrocodone Bitartrate/ibuprofen
Acetaminophen/propoxyphene Hydrochloride	Hydromorphone Hydrochloride
Acetaminophen/propoxyphene Napsylate	Ibuprofen/Oxycodone Hydrochloride
Acetaminophen/tramadol Hydrochloride	Ibuprofen/oxycodone Hydrochloride
Apap/butalbital/caff/codeine Phos	Levomethadyl Acetate Hydrochloride
Asa/oxycodone Hcl/oxycodone Terephthalate	Levorphanol Tartrate
Aspirin (buffered)/codeine Phosphate	Meperidine Hydrochloride
Aspirin/Butalbital/Caffeine/Codeine Phosphate	Methadone Hydrochloride
Aspirin/Carisoprodol/Codeine Phosphate	Morphine Sulfate
Aspirin/Codeine Phosphate	Morphine Sulfate/naltrexone Hydrochloride
Aspirin/butalbital/caffeine/codeine Phosphate	Naloxone Hydrochloride/pentazocine Hydrochloride
Aspirin/caffeine/dihydrocodeine Bitartrate	Oxycodone
Aspirin/caffeine/propoxyphene Hydrochloride	Oxycodone Hydrochloride
Aspirin/carisoprodol/codeine Phosphate	Oxymorphone Hydrochloride
Aspirin/codeine Phosphate	Propoxyphene Hydrochloride
Aspirin/hydrocodone Bitartrate	Propoxyphene Napsylate
Aspirin/oxycodone Hydrochloride	Tapentadol Hydrochloride
Aspirin/pentazocine Hydrochloride	Tramadol Hydrochloride
Belladonna Alkaloids/opium Alkaloids	
List of 15,299 NDC codes used to index prescription records available upon request.	

© 2022 Goldstick JE et al. *JAMA Network Open.*

eTable 3. ICD-9 & ICD-10 Codes Used to Indicate Clinical Characteristics

**Pain Category**	**ICD-9 Codes**	**ICD-10 Codes**
Osteoarthritis	715, 731, 732	M15, M16-M19, M89.4
Fibromyalgia	72.91	M79.7
Back/Neck Pain	720-723, 724, 737, 738.2, 738.4-738.5, 739.1-739.3	A18.01, M08.1, M25.78, M40-M41, M43.0-M43.2, M43.6, M43.8-M43.9, M45, M46.0-M46.1, M46.4-M46.5, M46.8-M46.9, M47, M48.0-M48.3, M48.8-M48.9, M49-M51, M53, M54, M62.830, M67.88, M95.3, M96.1-M96.5, M99.01-M99.03, M99.2-M99.7, M99.81, M99.83-M99.84
Non-Migraine Headache	307.81, 339, 784.0	G44, R51
**Covariates**	**ICD-9 Codes**	**ICD-10 Codes**
Substance Use Disorder	303, 304, 305.0, 305.2-305.9	F101, F102, F111, F112, F121, F122, F131, F132, F141, F142, F151, F152, F161, F162, F181, F182, F191, F192
Mood Disorder	296, 300.4, 311	F30-F39
Anxiety	300.0	F41, F42

© 2022 Goldstick JE et al. *JAMA Network Open.*

eTable 4. Percent Receiving Non-Opioid Pain Medication Prescriptions During Follow-Up in Each Cohort, and Stratified by Demographic Characteristics; in the Post-Guideline Period (2016-2018) and Differences Between the Observed Rate and That Predicted From the Pre-Guideline Trend

		Gender		Race		Insurance	
	Total	F	M	White	Other	Commercial Insurance	Medicare Advantage
Cohort 1	1,184,601 (20.6%)	734,800 (24.2%)	449,554 (16.6%)	795,152 (20.6%)	308,428 (22.1%)	843,533 (19.4%)	341,068 (24.3%)
Cohort 2	1,225,256 (20.6%)	758,639 (24.3%)	466,388 (16.6%)	814,817 (20.5%)	327,395 (22.2%)	821,671 (19.0%)	403,585 (25.1%)
Cohort 3	1,101,489 (20.4%)	676,587 (24.1%)	424,721 (16.4%)	727,517 (20.2%)	296,679 (22.1%)	706,482 (18.4%)	395,007 (25.1%)
Cohort 4	1,135,525 (20.1%)	696,537 (23.7%)	438,851 (16.2%)	725,929 (19.6%)	317,292 (22.0%)	711,065 (18.0%)	424,460 (25.0%)
Cohort 5 (actual-pred)	1,280,462 (20.8%) *+0.8%*	791,252 (24.4%) *+0.8%*	489,116 (16.8%) *+0.7%*	812,786 (20.2%) *+0.8%*	359,829 (22.9%) *+0.9%*	762,346 (18.5%) *+1.0%*	518,116 (25.5%) *+0.1%*
Cohort 6 (actual-pred)	1,514,936 (22.0%) *+2.1%*	943,115 (25.9%) *+2.5%*	571,768 (17.6%) *+1.6%*	937,860 (21.4%) *+2.3%*	434,711 (24.9%) *+2.9%*	798,406 (18.3%) *+1.3%*	716,530 (28.2%) *+2.6%*
Cohort 7 (actual-pred)	1,687,585 (22.2%) *+2.5%*	1,050,565 (26.0%) *+2.8%*	636,968 (17.8%) *+1.9%*	1,003,476 (21.6%) *+2.8%*	473,690 (25.3%) *+3.4%*	848,816 (18.4%) *+1.8%*	838,769 (28.0%) *+2.1%*

Notes: (actual-pred) are the observed levels minus the linear prediction; all entries are statistically significantly different from 0% at $p<0.05$.

Each cell shows counts receiving non-opioid pain medication prescriptions in each cohort, and in each group shown; parenthetical percentages underneath each count show the percent of that group, in that cohort, that received non-opioid pain medication prescriptions (e.g., 23.9% underneath 659,738 means that 659,738 comprised 23.9% of female patients in the 2012 cohort).

The “Other” Race category does not include those with Race labeled as “Unknown”, which was approximately 10% of all values, across years.

There were 4,215 missing values in the gender variable across years; those were in include in the other columns, but neither gender column.

© 2022 Goldstick JE et al. *JAMA Network Open.*

eTable 5. Percent Receiving Non-Opioid Pain Medication Prescriptions During Follow-Up in Each Cohort, and Stratified by Baseline Clinical Characteristics; in the Post-Guideline Period (2016-2018) and Differences Between the Observed Rate and That Predicted From the Pre-Guideline Trend

		Chronic Pain		Opioid Exposure		Substance Use Disorder		Anxiety Disorder		Mood Disorder	
	Total	Yes	No	Yes	No	Yes	No	Yes	No	Yes	No
Cohort 1	1,184,601 (20.6%)	384,718 (40.4%)	799,883 (16.7%)	489,248 (36.9%)	695,353 (15.7%)	30,005 (38.6%)	1,154,596 (20.3%)	134,638 (37.1%)	1,049,963 (19.5%)	201,306 (42.2%)	983,295 (18.6%)
Cohort 2	1,225,256 (20.6%)	415,415 (40.5%)	809,841 (16.5%)	506,801 (37.4%)	718,455 (15.7%)	34,078 (39.3%)	1,191,178 (20.3%)	152,708 (37.2%)	1,072,548 (19.4%)	215,881 (42.2%)	1,009,375 (18.6%)
Cohort 3	1,101,489 (20.4%)	379,321 (40.4%)	722,168 (16.2%)	455,024 (37.9%)	646,465 (15.4%)	34,802 (40.5%)	1,066,687 (20.0%)	147,700 (37.5%)	953,789 (19.0%)	202,773 (42.2%)	898,716 (18.2%)
Cohort 4	1,135,525 (20.1%)	387,469 (39.2%)	748,056 (16.1%)	451,057 (37.7%)	684,468 (15.4%)	39,379 (40.3%)	1,096,146 (19.7%)	157,611 (36.5%)	977,914 (18.7%)	211,193 (41.1%)	924,332 (18.0%)
Cohort 5 (actual-pred)	1,280,462 (20.8%) *+0.8%*	455,480 (40.1%) *+1.0%*	824,982 (16.4%) *+0.6%*	503,463 (40.0%) *+1.9%*	776,999 (15.9%) *+0.7%*	50,362 (42.6%) *+1.4%*	1,230,100 (20.4%) *+0.8%*	199,347 (37.7%) *+1.0%*	1,081,115 (19.2%) *+0.7%*	248,365 (42.2%) *+1.0%*	1,032,097 (18.6%) *+0.8%*
Cohort 6 (actual-pred)	1,514,936 (22.0%) *+2.1%*	578,409 (43.0%) *+4.2%*	936,527 (16.9%) *+1.3%*	606,254 (42.8%) *+4.4%*	908,682 (16.6%) *+1.5%*	66,623 (45.8%) *+4.0%*	1,448,313 (21.5%) *+2.1%*	275,392 (39.9%) *+3.4%*	1,239,544 (20.0%) *+1.7%*	310,155 (44.6%) *+3.7%*	1,204,781 (19.4%) *+1.9%*
Cohort 7 (actual-pred)	1,687,585 (22.2%) *+2.5%*	668,410 (42.7%) *+4.3%*	1,019,175 (16.9%) *+1.5%*	644,984 (43.9%) *+5.1%*	1,042,601 (17.0%) *+2.0%*	77,721 (45.6%) *+3.2%*	1,609,864 (21.6%) *+2.5%*	324,478 (39.6%) *+3.3%*	1,363,107 (20.1%) *+2.1%*	360,477 (44.3%) *+3.8%*	1,327,108 (19.5%) *+2.2%*

Notes: (actual-pred) are the observed levels minus the linear prediction; all entries are statistically significantly different from 0% at $p<0.05$.

Each cell shows counts receiving non-opioid pain medication prescriptions in each cohort, and in each group shown; parenthetical percentages underneath each count show the percent of that group, in that cohort, that received non-opioid pain medication prescriptions (e.g., 23.9% underneath 659,738 means that 659,738 comprised 23.9% of female patients in the 2012 cohort).

© 2022 Goldstick JE et al. *JAMA Network Open.*

Opioid Exposure is defined as having no opioid prescription claims during the 12-month baseline period for that cohort, and Chronic Pain is defined as having no claims history indicating chronic pain in the past year.

© 2022 Goldstick JE et al. *JAMA Network Open.*

eFigure 1. Schematic Diagram of the Sequential Cohorts

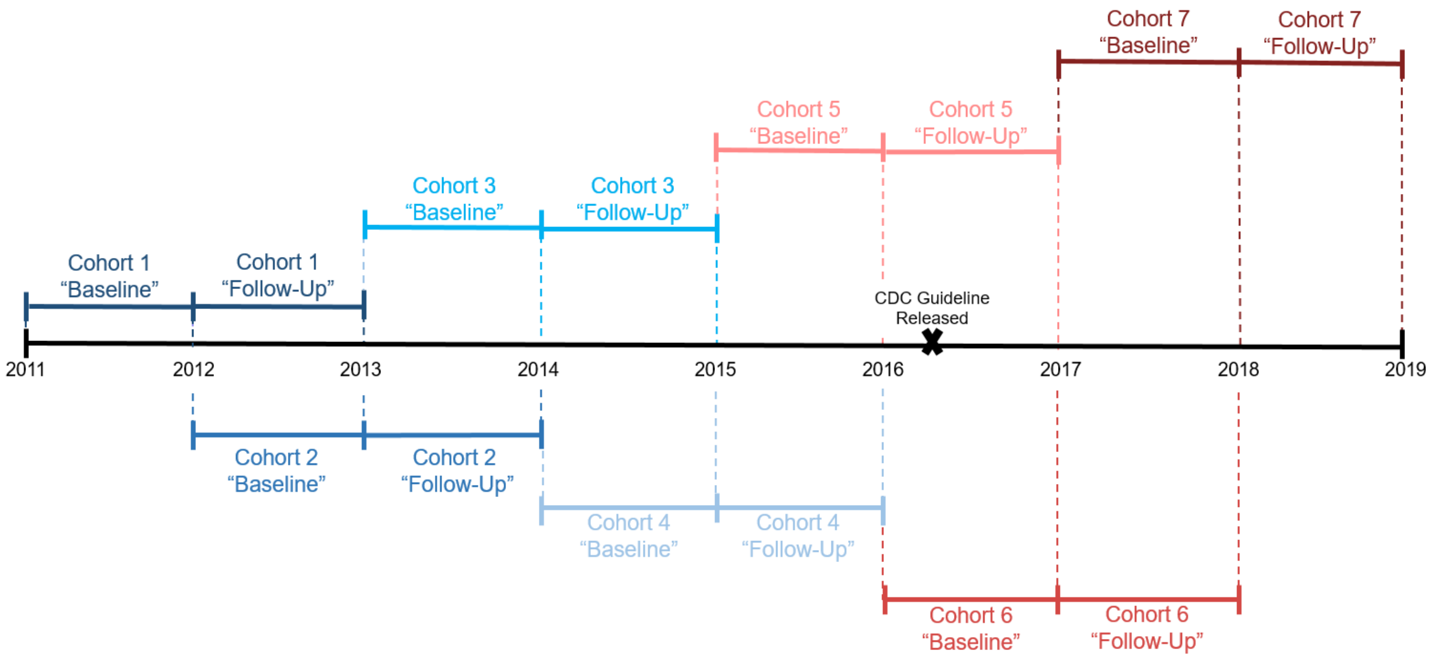

© 2022 Goldstick JE et al. *JAMA Network Open.*

eFigure 2. Total Number Eligible for Each Cohort, and How Many Were Deleted Due to the Continuous Enrollment Requirement, the Age 18+ Requirement, and the Cancer Exclusion

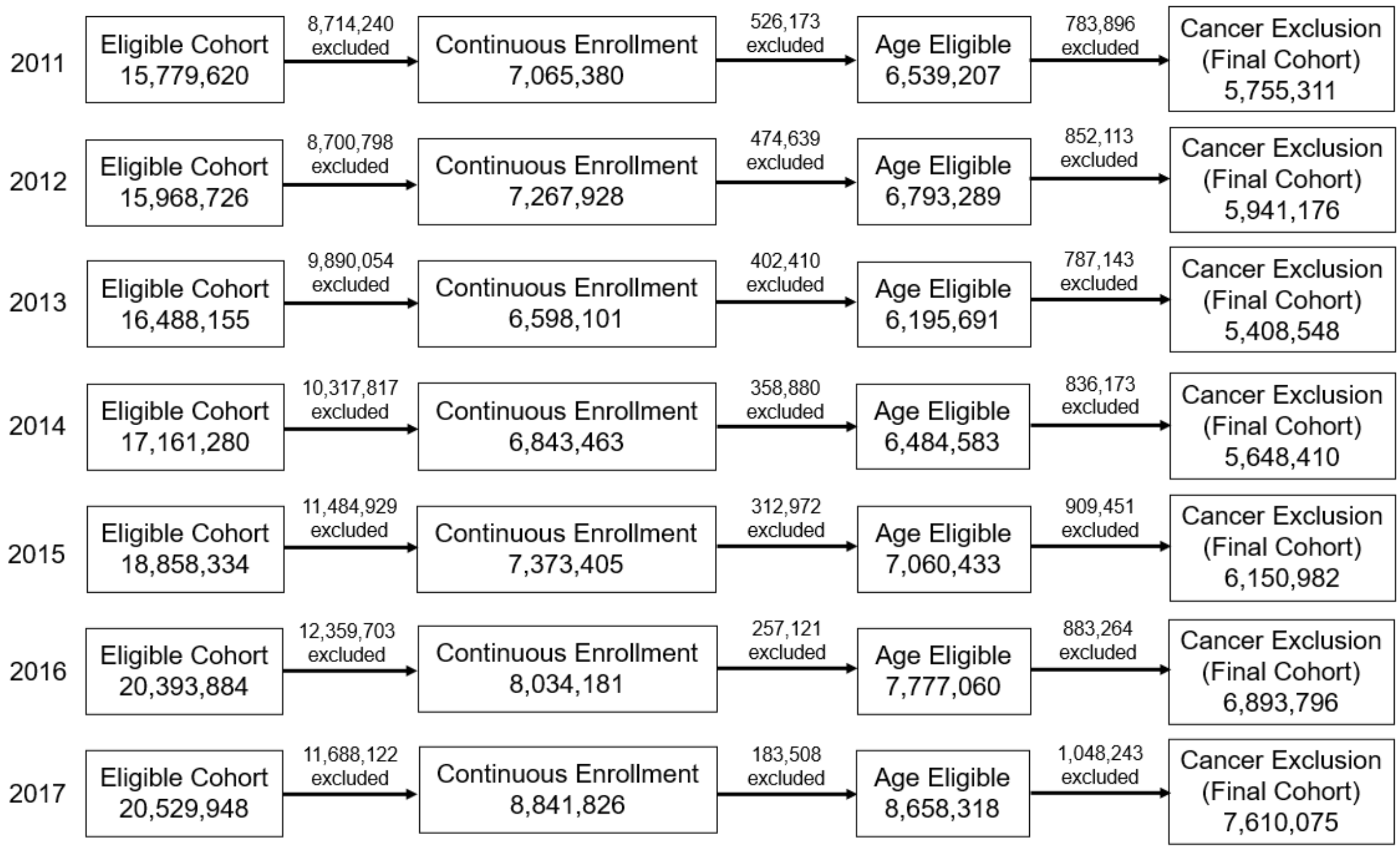

© 2022 Goldstick JE et al. *JAMA Network Open.*
